# *Hydrophylita (Lutzimicron) emporos* Shih & Polaszek (Hymenoptera: Trichogrammatidae) from Taiwan, Parasitising Eggs, and Phoretic on Adults, of the Damselfly *Psolodesmus mandarinus mandarinus* (Zygoptera: Calopterygidae)

**DOI:** 10.1371/journal.pone.0069331

**Published:** 2013-07-24

**Authors:** Yuan Tung Shih, Chiun Cheng Ko, Kuang Tao Pan, Sue Cheng Lin, Andrew Polaszek

**Affiliations:** 1 Department of Entomology, National Taiwan University, Taipei, Taiwan; 2 Endemic Species Research Institute, Taichung, Taiwan; 3 Department of Life Sciences, Natural History Museum, London, United Kingdom; 4 Imperial College of Science, Technology and Medicine, London, United Kingdom; University of Sussex, United Kingdom

## Abstract

*Hydrophylita emporos* n. sp. reared from eggs of *Psolodesmus mandarinus mandarinus* McLachlan (Zygoptera: Calopterygidae) in Taiwan is described. This is the first species of *Hydrophylita* to be described from the Old World, and the first record of phoresy in the genus. Adult females were observed aggregating at the base of the female damselfly’s abdomen. When the damselfly begins ovipositing, females move to the tip of the abdomen, enter the water and quickly locate eggs for parasitising. The article contains links to video footage of this process.

## Introduction

Trichogrammatidae is one of the least-studied families of Chalcidoidea, mainly due to their small size, down to 0.17 mm in the genus *Megaphragma*, making them the smallest fully-winged insects. The exception is the speciose and relatively well-studied genus *Trichogramma*, many species of which are used widely as biological control agents of pest insects [1], [2], mostly Lepidoptera. The family currently contains about 800 species in 84 genera worldwide [3]. Three genera, *Hydrophylita*, *Lathromeroidea* and *Prestwichia*, are known to parasitise eggs of aquatic insects. There are four described *Hydrophylita* species, all from the New World, although Pinto [3] recorded undescribed species from Australia, Indonesia and Madagascar. The nominal subgenus is known so far only from the New World, while *Lutzimicron* occurs in both the Old and New Worlds.

Available host records for *Hydrophylita* species were reviewed by Querino and Pinto [4], and comprise the Zygopteran families Coenagrionidae (*Ischnura verticallis*) and Lestidae (*Lestes* sp.) [5], [6]. The present record is the first from the Calopterygidae. *Hydrophylita emporos* is a parasitoid of *Psolodesmus mandarinus mandarinus* McLachlan. Little is known about the taxonomy, biology and life cycle of aquatic egg parasitoids in Odonata, but Clausen [7] stated that some trichogrammatid adults are adapted to an aquatic environment, and are capable of either swimming or entering the water by crawling down plant stems or other objects. During the present study, females of *H. emporos* were observed to be phoretic on adult damselflies, with several often being found at the base of the damselfly’s abdomen. Their observed behavior is described in detail below.

## Materials and Methods

Field collection and observation were carried out at the study site of Alibang, Shimen District, New Taipei City. This abandoned tea farm, now completely under secondary forest, is not in a national park or protected area, and thus no specific permission is required (no collecting permits are needed). No endangered or protected species were involved in this study. Eggs of *Psolodesmus mandarinus mandarinus* embedded in submerged leaves of *Piper kadsura* plant tissue were brought to the laboratory for parasitoid rearing, although a proportion of leaves were maintained in plastic cages in the river for a few days. In order to prevent bacterial infection the leaves in the laboratory were maintained in containers with twice-distilled water. After 3–5 days eggs were removed from the decaying leaves, and separated into fresh containers. Dishes were covered to prevent any emerging wasps from escaping. Emerging individual parasitoids were maintained at room temperature (22±2°C), 85–95% RH and natural photoperiodic cycle. Specimens were photographed with an electronic eyepiece digital camera attached to a stereomicroscope Leica Zoom 2000, and illustrated using a light microscope Olympus BX51 in the Dept of Entomology, National Taiwan University, Taiwan.

Specimens comprising the type series were first treated with Proteinase K for DNA extraction following a standard non-destructive protocol developed at the Natural History Museum, London (NHM). They were then slide-mounted in Canada balsam using a standard procedure modified from Noyes [8].

Polymerase Chain Reaction (PCR) was undertaken for mitochondrial CO1 and ribosomal 28S D2 gene fragments using the following primer pairs:

28S D2 foward

D23f 5′ - GAGAGTTCAAGAGTACGTG

28S D2 reverse

28SRev 5′ - TTGGTCCGTGTTTCAAGACGG

CO1 forward

1FCO1 5′ - GGAGGATTTGGAAATTGRYTWRTTCC

CO1 reverse

1RCO1 5′ - ACTGTAAATATRTGATGWGCTCA

DNA sequencing was carried out for these gene fragments at the NHM. Resulting sequences were analysed and edited using Sequencher version 4.8. Identical sequences were obtained for 5 individuals for both genes, and these have been deposited in Genbank under accession nos KF053530 (CO1) and KF053531 (28S), respectively.

Morphological terminology and the format for species descriptions follow Doutt and Viggiani [9]. Terms and acronyms associated with the antennal sensilla are derived from those used for *Trichogramma* [3], [4], [10], [11]. Antenna: basiconic peg sensilla (BPS); placoid sensilla (PLS); aporous sensillar trichodea B (socketed) (APB); aporous seta A (APA); flagelliform setae or multiporous pitted sensilla trichodea A (unsocketed) (FS); recurved sensilla (RS); uniporous pit pore sensilla trichodea D (UPP). Fore wing: premarginal (PM) ( = parastigma), marginal (MV) and stigmal (SV) veins; wing length (FWL), wing width (FWW). For females: ovipositor length (OL), hind tibia length (HTL). All measurements are given in millimetres.

### Nomenclatural Acts

The electronic edition of this article conforms to the requirements of the amended International Code of Zoological Nomenclature, and hence the new names contained herein are available under that Code from the electronic edition of this article. This published work and the nomenclatural acts it contains have been registered in ZooBank, the online registration system for the ICZN. The ZooBank LSIDs (Life Science Identifiers) can be resolved and the associated information viewed through any standard web browser by appending the LSID to the prefix "http://zoobank.org/". The LSID for this publication is:

urn:lsid:zoobank.org:pub:9CCC051E-FFC6-439F-8E3F-7FB2ADAED980.

The electronic edition of this work was published in a journal with an ISSN, and has been archived and is available from the following digital repositories: PubMed Central, LOCKSS.

### Genus *Hydrophylita* Ghesquière

*Hydrophylita* Ghesquière 1946: 371 [12]. Type species: *Hydrophylax aquivolans* Matheson and Crosby, 1912 [13]. replacement name for *Hydrophylax* Matheson & Crosby, 1912: 65 [13]; *Lutzimicron* Costa Lima, 1960: 197 [6].

### Diagnosis

For a full generic description of *Hydrophylita* and a discussion of morphological characters of *Hydrophylita* and closely related genera see Pinto (2006: 64–65). The presence of the following character states is required for a positive diagnosis of *Hydrophylita* (females): Antenna with 1 or 2 anelli, 2 funicular and usually 3 claval antennomeres; all postanellar antennomeres longer than wide; PLS absent from funicle; BPS elongate, narrow, often apically attenuate; apex of clava with two large unsocketed spatulate sensilla. Mandible with a large posterior spine. Maxillary palp with 1 or 2 palpomeres. Mid lobe of mesoscutum and scutellum each with 2 pairs of setae. Propodeum elongate and longer than scutellum. Fore wing narrow; disk with densely setose.

Revisionary studies of *Hydrophylita* for the New World are as follows: North America: [13] (as *Hydrophylax*)]; South America: [3], [4], [6], [14].

**Figure 1:**
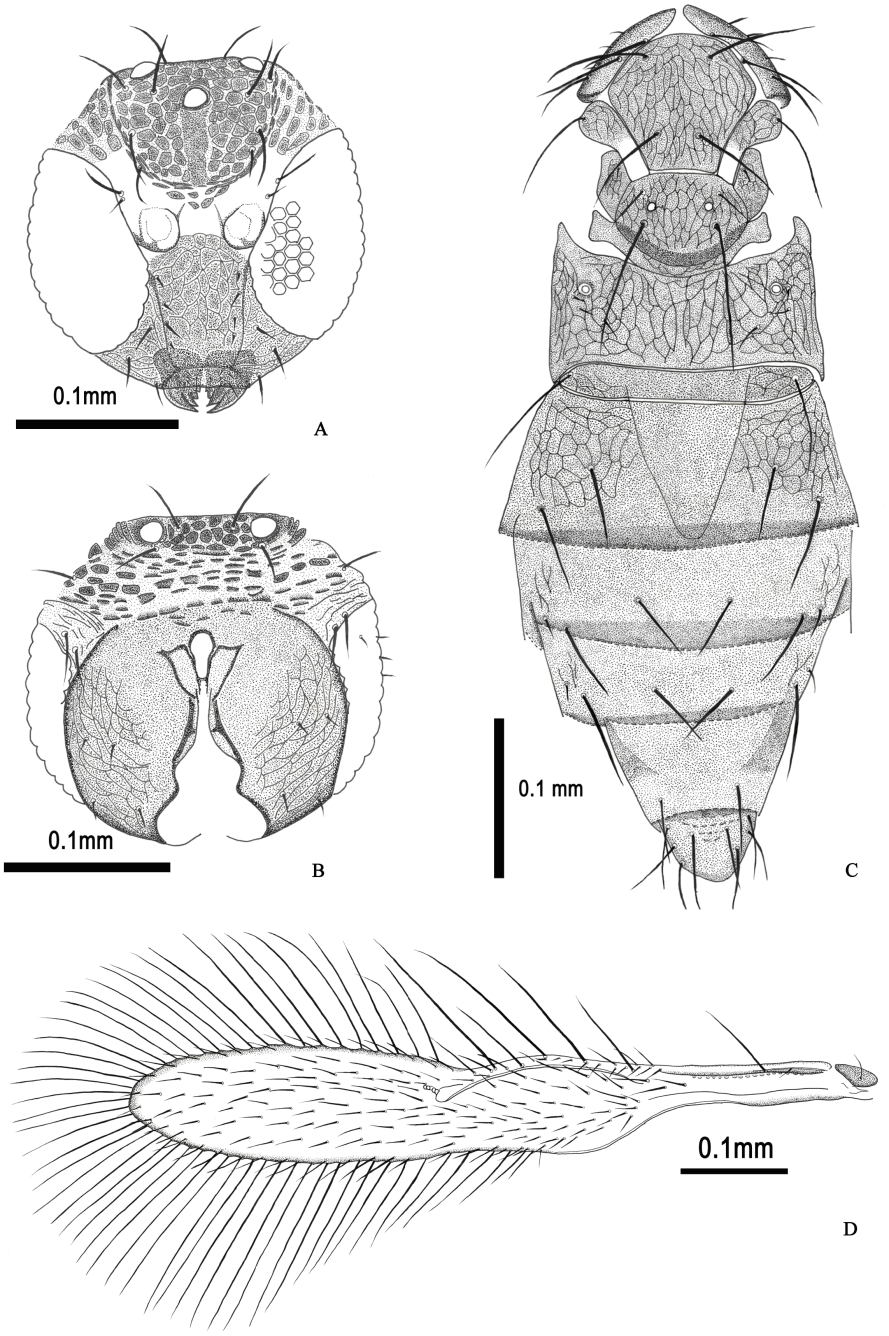
*Hydrophylita emporos* n. sp., female. A. anterior head. B. posterior head. C. mesosoma and metasoma. D. fore wing.

**Figure 2:**
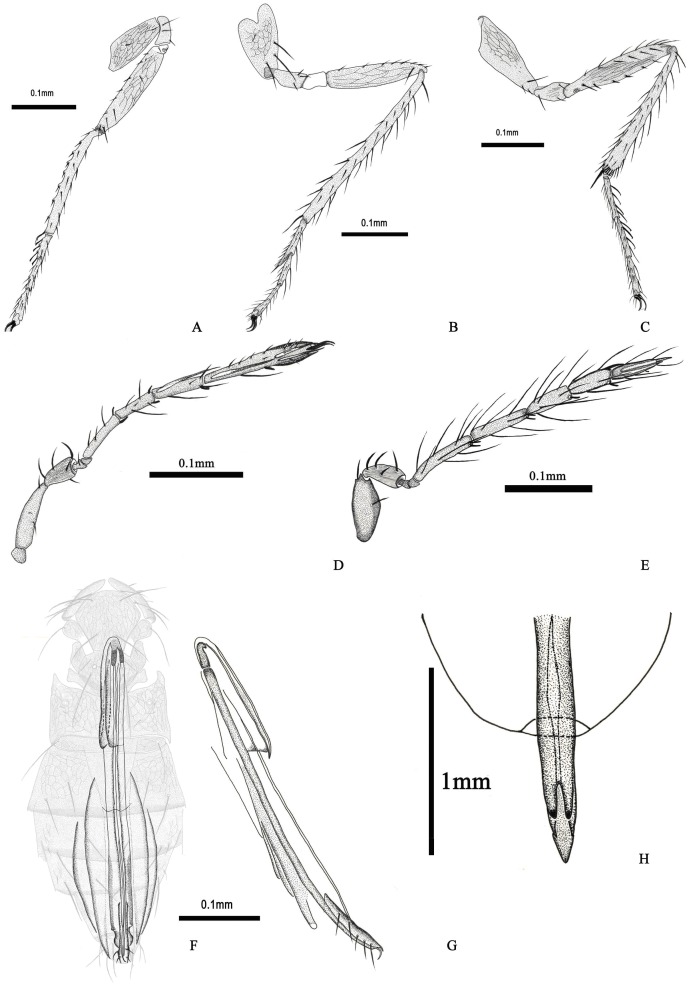
*Hydrophylita emporos* n. sp., female and male. A. front leg. B. mid leg. C. hind leg. D. female antenna. E. male antenna. F. ovipositor relative to body. G. ovipositor. H. male genitalia.

**Figure 3:**
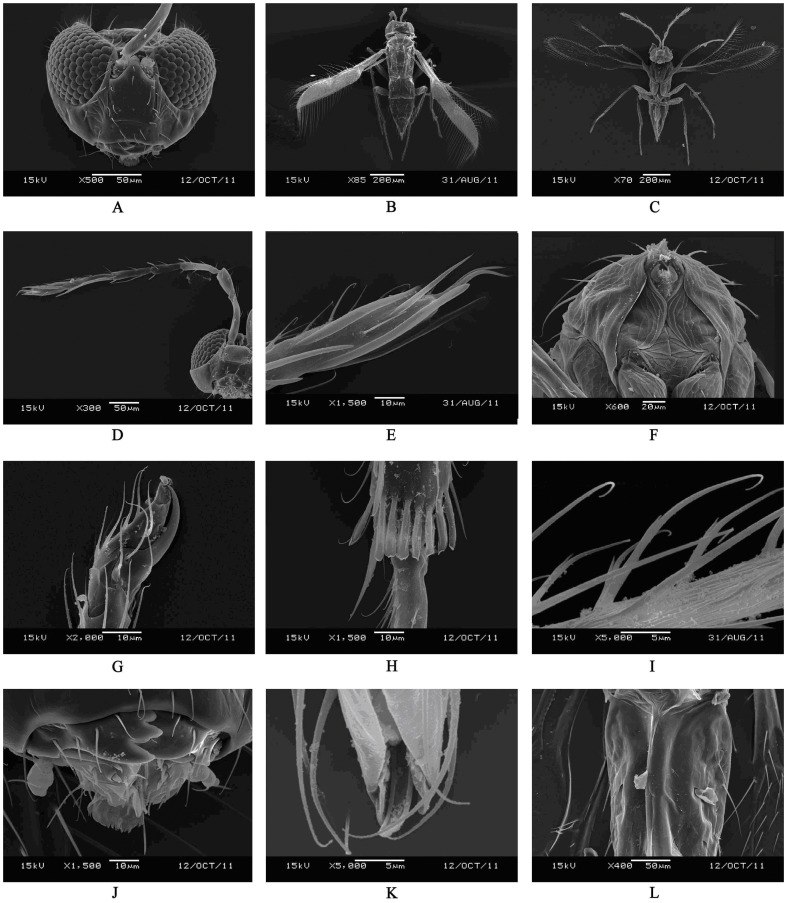
Scanning electron micrographs (SEM) of *Hydrophylita emporos* n. sp., female. A. head. B. dorsal view of adult. C. ventral view of adult. D. antenna. E. propleura and prosternum. F. apex of second claval antennomere. G. claws. H. spatulate structures of hind tibia. I. setae of midtibia. J. mandibles (arrow indicates teeth of galea). K. apex of ovipositor. L. second valvifer (excluding legs).

**Figure 4:**
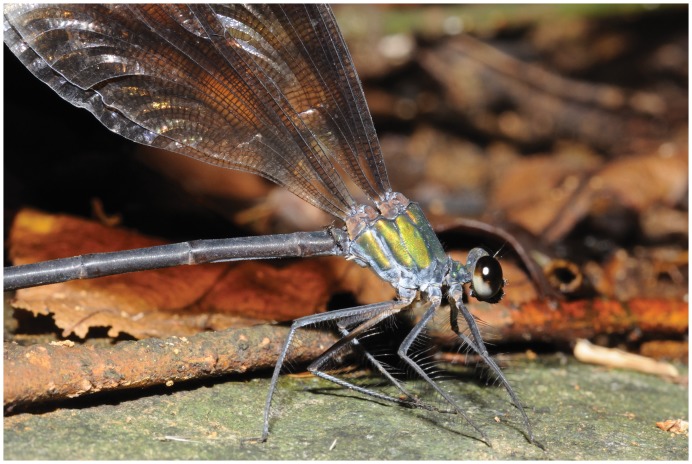
*Psolodesmus mandarinus mandarinus* with female of *Hydrophylita emporos* phoretic on base of abdomen.

**Figure 5:**
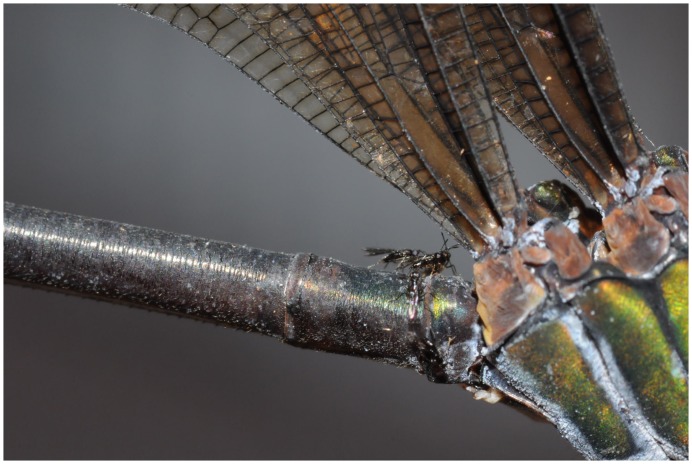
Detail of *Psolodesmus mandarinus mandarinus* with female of *Hydrophylita emporos* phoretic on base of abdomen.

**Figure 6:**
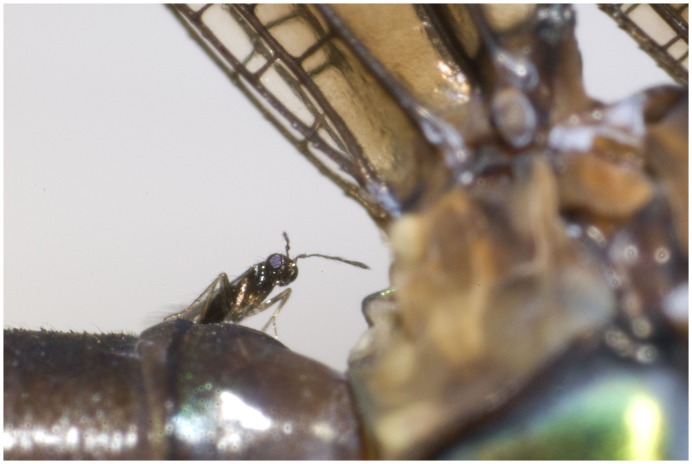
Detail of *Psolodesmus mandarinus mandarinus* with female of *Hydrophylita emporos* phoretic on base of abdomen.

**Figure 7:**
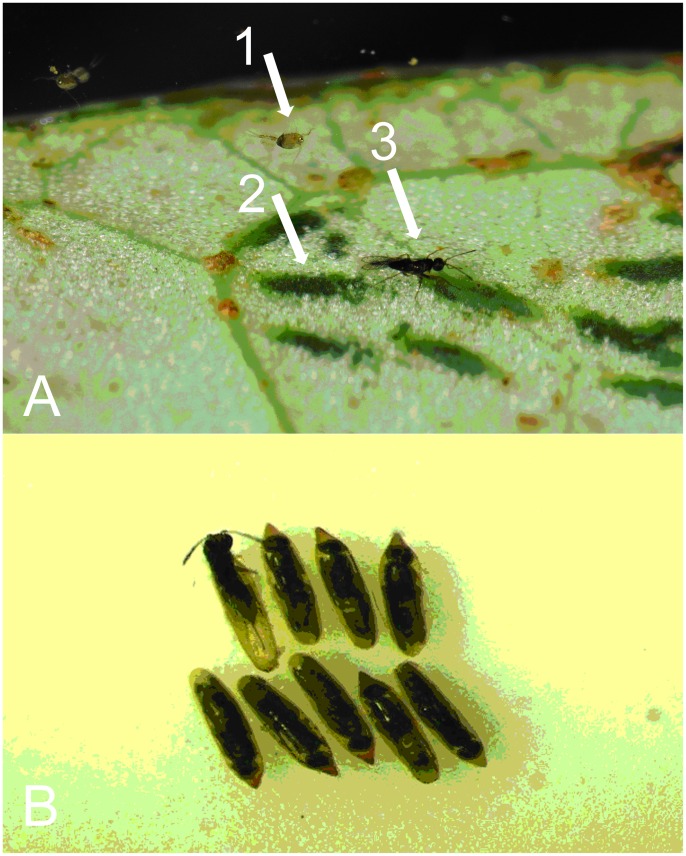
*Psolodesmus mandarinus mandarinus* under water in the field (arrow 1 indicates a water flea; arrow 2 indicates the damselfly eggs; arrow 3 indicates *Hydrophylita emporos*). B. emergence of adults.

***Hydrophylita*** **(** ***Lutzimicron*** **)** ***emporos*** **Shih and Polaszek n. sp.**

(Figs 1, 2, 3, 4, 5, 6, 7).

urn:lsid:zoobank.org:act:DAD240DB-E61E-46B4-AACD-728AD5D56D90.

### Description

*Female:* length 1.1–1.2 mm.

#### Colour

Head dark brown except pale brown area present around stemmaticum. Mesosoma and metasoma dark brown except the basal area of side lobes of mesoscutum pale yellow. Antenna pale brown. Fore wing hyaline, but with a distinct dark spot at distal end of marginal vein extending below to stigmal vein. Legs pale brown except trochantellus of front and mid legs. Second valvifer and apex of third valvula dark brown.

#### Morphology

Body shape slender, with long setae (Figs 3B, C). Antenna with 8 antennomeres, elongate, narrow. Two anelli (A) present, funicle (F) with two antennomeres, clava (C) with two antennomeres (C1 and C2), C2 and C3 fused completely (Fig. 2D); A1 cupuliform; scape four times as long as wide, and longer than pedicel (1.86); F1 1.21–1.35× length of F2, F2 subequal in length to C1; C2 distinctly longer than C1 (2.26), C2 also longer than each funicule. Antenna with scattered setiform sensilla (Fig. 3D) and fusiform BPS, distribution and types of sensilla by antennomere as follows: Radicle–5 APA (the setae surround the base of radicle); Scape –3 APA; Pedicel –5 APB; A1–1 APB; A2–0; F1–3–5 APB, 2 BPS; F2–5–8 APA or APB, 1 FS subequal in length to F2, 2 BPS; C1–4–7 APA or APB, 2 FS, 1–2 BPS, 1 PLS 1.26–1.3× length of C1; C2–11–14 APA or APB, 1FS 0.38–0.41× length of C2, 1–2 BPS, 5 PLS (1 entirely free of surface, 2 appressed along entire length except apex which extends slightly beyond tip of antennomere, and 2 appressed only at basa), 3 subapical conelike sensilla, 2 spinelike RS (UPP at very apex of antennomere) (Fig. 3E).

Head 1.07–1.2× as wide as long, 1.4× as wide as mesosoma (Fig. 3A). Eye with dense setae, especially close to the vertex. Stemmaticum with tessellated surface sculpture, and with 12–14 setae. Frons with 14–16 setae, mostly close to gena. Postgena with 8 setae, vertex with 2 strong setae in each side. Clypeus with 4–8 setae (Figs 1A, B). Mandible with 5 teeth (1 distinct anterior socketed tooth) and 4 sensilla. Two maxillary palpomeres, second with 2 setae at apex, galea with 5 teeth (Fig. 3J).

Mesosoma. Pronotum with 3 long and 3 short setae (Figs 1C, 3C). Mid lobe of mesoscutum 1.12× as wide as long, with two pairs of elongate setae, subequal to the length of mid lobe; side lobe of mesoscutum with 1 long seta each, 1.29–1.35× length of side lobe, surface reticulate; scutellum similarly sculptured and with two pairs of elongate setae, posterior pair the longest on mesosoma, and subequal in length to the width of mid lobe, and 2.86–3.12× length of anterior pair, distance between bases of anterior scutellar setae slightly more than that of between posterior pair; scutellar sensilla widely placed, separated by 7× maximum width of a sensillum (Fig. 1C). Axillae large, trapezoidal, and projecting forwards, with 1 short seta each. Propodeum large, elongate, projecting forward laterally, and with 3 pairs of setae. Propodeum 1.78–1.83× and 6.25–7.8× scutellar and metanotal length, respectively. Petiole with one pair of elongate setae laterally, 0.82× length of scutellar posterior setae. Fore wing relatively narrow, subspatulate, 7× as long as wide, narrowly rounded at apex (Figs 1D, 3B); membrane densely setose, setae moderately elongate. 5–8 long setae basally; fringe setae elongate on all margins, those on antero– and posteroapical margins subequal, the length of the longest fringe setae (FrS) occur on the posteroapical margin, 1.5–1.85× FWW; venation extending 0.5× FWL, PM and MV confluent, MV with 3 distinct, long setae, 1.3–1.45× FWW, and MV 1.29–1.35×length of SV; PM with 2 setae, posterior seta elongate, 5.4–6.2× anterior length; SV slightly and gradually widening to apex, without a distinct stigma (Fig. 1D). Hind wing elongate, narrow, 19.1–21× as long as wide, with 3 complete setal tracks, posterior fringe setae considerably longer than those on anterior margin, 4.2× wing width. All legs densely setose, some setae on femur and tibia fork-like (Fig. 3I). Fore tibia with 7 thorn–like setae on the dorsal surface, apical spur of fore tibia barb–like, basitarsus with 4 spatulate structures on the dorsal surface, coxa and femur reticulate (Fig. 2A); Apical spur of mid tibia 0.42–0.56× the length of basitarsus, mid basitarsus with distal extension with apical seta; coxa with 3 long setae 1.28× length of midtibial spur, coxa and femur reticulate (Fig. 2B). Hind tibia 1.22–1.31× hind tarsal length, 3.9–4.2× length of first tarsomere, and with 8 spatulate structures (Figs 2C, 3H); tarsomeres of each leg subequal in length, except mid basitarsus; claws of each leg enlarged and strong (Fig. 3G).

Metasoma. Metasomal terga with following numbers of setae: T1: 4, T2: 6, T3: 6, T4: 2, T5: 8. Ovipositor relatively long, extending from the basal midlobe of mesoscutum to the apex of T5; apex of ovipositor hook–shaped (Fig. 3K), third valvula 1.6 times as long as second valvifer and with 4 setae in the apex, OL 1.45× length of gaster, OL/HTL (2.3–2.5) (Figs 2F, 2G, 3L).

#### Male

*Colour*. Very similar to that of female, the main difference being an entirely dark mesosoma and metasoma. *Morphology*. Antenna with 9 antennomeres, C2 and C3 separate (Figs 2E); scape wider than in female, 2.32× as long as wide; funicle longer than in female, 1.42× clava length, C1, C2 and C3 subequal in length, both funicular antennomeres considerably longer than any of the claval antennomeres (e.g. F1 1.5× length of C1); length/width of antennomeres: scape 2.32, pedicel 2.31, F1 6.1, F2 5.4, C1 3.3, C2 2.85, C3 3.63. Antenna with longer setiform sensilla and fusiform BPS than female, APA and APB absent from funicle and clava, distribution and types of sensilla by antennomere as follows: R–0; S–2 APA; P–4 APB; A1–0; A2–0; F1–8 FS (longest one 0.83× length of F1), 2 BPS; F2–6 FS, 2 BPS; C1–7 FS (longest 1.2× length of C1), 1 BPS, 0 PLS; C2–5FS (all FS subequal in length, 1.1× length of C2), 1 BPS, 1 PLS (appressed to antennomere along entire length except apex, which extends beyond tip to the basal part of C3); C3–5 FS longest subequal in length to C3, 1 BPS near the middle, 2 PLS (appressed to antennomere for their entire length, one PLS extends greatly beyond tip of antennomere, spinelike) (Fig. 2E). Genitalia with genital capsule (GC) relatively narrow (Fig. 2H), side of GC almost parallel; aedeagus slightly longer than GC (1.1× GL) and 0.76× HTL; apodemes comprising 0.8 AL; GC with VS extending 0.75 length of PAR; PAR and VS without any spines.

Holotype female: TAIWAN: Alibang, Shimen District, New Taipei City 25°16′N 121°34′E (Y.T. Shih & S.C. Lin col) 27.viii.2011, ex egg *Psolodesmus mandarinus mandarinus* on *Piper kadsura* (National Taiwan University, Taipei, Taiwan, Arthropod Museum). Paratypes: 6 females on 6 slides, same data as holotype (BMNH); 11 females, 1 male, same data as holotype but 20.vii.2011 (National Taiwan University, Taipei, Taiwan, Arthropod Museum: 4; Natural History Museum, London, UK.: 3; Endemic Species Research Institute, Taichung, Taiwan: 2; Taiwan Agricultural Research Institute, Taichung, Taiwan: 1).

### Etymology

The specific epithet *emporos* means “passenger” in Latin, reflecting the phoretic behaviour of adult females.

### Host

Eggs of *Psolodesmus mandarinus mandarinus* McLachlan (Zygoptera: Calopterygidae).

### Molecular Studies

The 28S D2 sequences were all of a very high quality, with almost no ambiguous sites. Blasting the sequence in Genbank (Accession no. KF053531) gave a very close match (96%) with an existing sequence of an undescribed *Hydrophylita* resulting from the work of Gillespie *et al* [15]. Conversely, the CO1 sequence (Genbank Accession no. KF053530) when blasted gave no close matches, giving either several *Eusandalum* sequences (Chacidoidea: Eupelmidae) resulting from the work of Althoff [16], or *Cecidostiba fungosa* (Chacidoidea: Pteromalidae) resulting from the work of Kaartinen *et al*. [17], depending on which blast options were used.

### Remarks

Of the described *Hydrophylita* species, *H. emporos* appears to be most closely related to *H. lestesi* Costa Lima [6], but differs from it in having the female antenna with only two claval antennomeres, with C2 and C3 completely fused. The fore wing is 7× as long as wide and the ovipositor extremely elongate (much longer than the combined length of scutellum, propodeum and metasoma). Currently, *H*. *emporos* is the only species of *Hydrophylita* described from the Old World, although Pinto [3] recorded undescribed species from Australia, Madagascar and Indonesia.

Key to the species of female *Hydrophylita* (modified from Querino and Pinto [4]).

1. Fore wing very narrow with apex distinctly pointed; its length at least 14× its width, disk with only a single line of setae. Antenna with placoid sensilla (PLS) on each claval antennomere attached to surface almost their entire length…subgenus *Hydrophylita*.2
  Fore wing wider, with apex slightly pointed; its length less than 10× its width, disk densely setose (Fig. 1D). Antenna with one or more PLS on each claval antennomere spinelike, attached to surface only at the base (Fig. 2D, E)…subgenus *Lutzimicron*.3
2. Antenna with one anellus (North America)…*H. aquivolans*
  Antenna with two anelli (Fig. 3B) (Central and South America)…*H. bachmanni*
3. Antenna with 7 antennomeres, C2 and C3 completely fused. (Fig. 2D) (Asia)…*H. emporos* sp. n.
  Antenna with 8 antennomeres, C2 and C3 separate…4
4. Antenna with funicular antennomeres approximately equal in length, first claval antennomere (C1) approximately equal in length to first funicular antennomere (F1); clava poorly differentiated from funicle. Fore wing 8× as long as wide (Brazil)…*H. lestesi*
  Antenna with F2 and C1 both distinctly shorter than F1, clava well differentiated from funicle. Fore wing 6× as long as wide (broadly distributed in Neotropics)…*H. neusae*

## Discussion

The damselfly species, *P*. *mandarinus* includes three subspecies: *P*. *mandarinus mandarinus* McLachlan, 1870 from northern Taiwan, *P*. *mandarinus dorothea* Williamson, 1904 from southern Taiwan and *P*. *mandarinus kuroiwae* Matsumura, 1913 from Japan. *P*. *mandarinus mandarinus* is widely distributed in northern parts of Taiwan and its population peaks during May to October. The parasitoids were found in North Taiwan only, whereas no infestation was observed in other subspecies found in the southern part of the island. *P*. *mandarinus mandarinus* uses its ovipositor to cut a hole for laying eggs on leaves of *Piper kadsura* plants submerged in water. All the members of the plant family Piperaceae are climbers, and are usually found above ground level. However, some plants grow near water, and branches often fall into the water, and *P*. *mandarinus* lay eggs on these submerged parts.

On many occasions, one or more *H. emporos* was observed positioned on the base of the damselfly abdomen, awaiting the damselfly’s act of oviposition (Figs 4, 5, 6). Once the damselfly starts probing below the surface of the water before laying eggs, the parasitoids walk rapidly along the abdomen towards its distal end. Female wasps then appear to take advantage of the abdomen breaking the water surface in order to more easily enter the water (Fig. 7, and video footage at http://vimeo.com/59398646) in order to parasitise the freshly-laid damselfly eggs. Female *H*. *emporos* use their legs and wings to walk and/or swim under water freely, to reach leaves and begin to parasitize eggs. Occasional observations of *H. emporos* females apparently struggling to submerge without the help of the damselfly abdomen as a substrate, support the theory that the use of the damselfly abdomen greatly facilitates entry into the water. However, we have no evidence that phoresy is obligatory in this species.

Most adult females of *H*. *emporos* emerge under water and then swim to the surface, but some apparently choose to stay under water for their entire life cycle. Eggs that remained on the leaves for two weeks gave rise to higher levels of parasitoid mortality, though no quantitative assessment was made. Video film of *H. emporos* can be found at: http://vimeo.com/59398646.

Males of *H. emporos* appear to be very rare (125 females for one male), and we believe that the males may remain under water for most of their life cycle. It is unknown how these parasitoids overcome the effect of the current of water while searching for hosts, and are apparently able to respire under water for up to 24 hours. Several morphological characters in females might be adaptations to an aquatic environment, for example the unusually enlarged claws that may enable them to climb and crawl along the river bed more easily. These observations are the first for any *Hydrophylita* species, and further study of *H. emporos* is currently being carried out to reveal specific morphological adaptations to a semi- or mainly aquatic life cycle in these very unusual parasitoid wasps.

Clausen [7] recorded phoresy by adult females prior to ovipositing on the eggs of their hosts in six families of Hymenoptera and one of Diptera, separating this type of phoresy from that involving transport of first instar larvae. One characteristic of the former type of phoresy is that all known species with this habit attack hosts that deposit their eggs in large masses, as in the present case. The great advantages of this kind of phoresy is that the host transports the parasitoid directly to the latter’s food source, and the host eggs are immediately accessible, and presumably at their most vulnerable before any or little development has occurred.

Phoresy is recorded here for the first time in *Hydrophylita*, but has been previously observed in several other genera of Trichogrammatidae. *Trichogramma dendrolimi* are known to be phoretic on the wings of the moth *Dendrolimus sibiricus* [7] and *T. brassicae* is known to ride on its host *Pieris brassicae* [18]. Two *Brachista* species are phoretic on robberflies [19], though it has not been established whether they are definite hosts. *Pseudoxenufens forsythi* is known to be phoretic on a number of nymphalid butteflies [19], [20], and finally two *Oligosita* species are phoretic on Orthoptera. [21].

## Supporting Information

Movie S1 (MOV)
