# Correction: *Hydrophylita (Lutzimicron) emporos* Shih & Polaszek (Hymenoptera: Trichogrammatidae) from Taiwan, Parasitising Eggs, and Phoretic on Adults, of the Damselfly *Psolodesmus mandarinus mandarinus* (Zygoptera: Calopterygidae)

**DOI:** 10.1371/annotation/cf1aea8b-a03c-46ab-9456-05d054a7243a

**Published:** 2013-12-16

**Authors:** Yuan Tung Shih, Chiun Cheng Ko, Kuang Tao Pan, Sue Cheng Lin, Andrew Polaszek

The first two authors, Yuan Tung Shih and Chiun Cheng Ko, should be indicated as having contributed equally to the article. 

